# Global Initiative of the Special Olympics Movement for People with Intellectual Disabilities

**DOI:** 10.1515/hukin-2015-0026

**Published:** 2015-04-07

**Authors:** Andrzej Myśliwiec, Mariusz Damentko

**Affiliations:** 1 Department of Kinesitherapy and Special Methods in Physiotherapy, The Jerzy Kukuczka Academy of Physical Education, Katowice, Poland.; 2 Special Olympics Europe- Eurasia, Warszawa.

**Keywords:** Special Olympics, sports, intellectual disabilities

## Abstract

The mission of the Special Olympics is to provide year-round sports training and competition in a variety (33) of Olympic-type sports for children and adults with intellectual disabilities, giving them continuing opportunities to develop physical fitness, demonstrate courage, experience joy, and participate in sharing of gifts and friendship with their families, other athletes, and their communities. The Special Olympics movement often goes beyond the sports competition formula. During the last few years, the movement has developed many new global initiatives, which expand its former sports activities. They include:

1. Coaching excellence and the coaching model
2. Partnerships with international (regional) sports federations
3. Sports Resources Teams (SRT)
4. Extended quota for high level athletes
5. Athletes Leadership Program (ALPS)
6. Young Athletes Program
7. Youth volunteer initiatives
8. Unified Sports Program
9. Motor Activity Training Program
10. Healthy Athletes Program

These initiatives fulfill and expand the existing program, which was launched in 1968 and is the largest sports organization for people with disabilities worldwide, with very important new social, marketing, and developmental aspects of life, going far beyond activities met in other sports organizations.

## Introduction

The mission of the Special Olympics (SO) is to provide year-round training and sports competitions in a number of sport disciplines for children and adults with intellectual disabilities (ID), giving them an opportunity for better development, to demonstrate courage, and experience joy of sharing achievements with friends, family, and their local communities. However, the SO movement has frequently gone beyond sport competitions. The SO was founded in 1968 in the United States by the initiative of Eunice Kennedy-Shriver. At present, it brings together over 5.2 million athletes in 177 countries and from all the continents who practice the 33 different sports disciplines (Special Olympics, 2014).

In 1971, the SO gained the right to use in their actions the word Olympics and to employ the ceremony of the Olympic Games, which includes the lighting of the Olympic flame and using the Olympic flag (Janusiewicz-Kuder et al., 1999).

SO competitors have the opportunity to compete in both summer and winter disciplines. The most popular sports include soccer, swimming, and track and field (Special Olympics, 2014). The conditions for beginning training and competing include being 8 years old, having an intellectual disability degree certificate and physician’s consent to participate in a particular sports discipline.

The unique feature of the SO is the commonness of the sports. In 2007, for example, there were about 20,000 sporting events in 30 winter and summer disciplines around the world and 200 competitions in 20 disciplines in Poland (Special Olympics Poland, 2014). The SO games have an extremely specific course in respect to rules. Athletes with different degrees of disability can participate in numerous events, and for the competition purposes the competitors are divided into ability groups. Each group may include no less than three and no more than eight participants, and the difference in the results obtained in the preliminary eliminations between the best and the weakest competitors in a group cannot be greater than 10 to 15%.

The SO have recently initiated numerous new, global initiatives that broaden their current activities. Our objective was to gather and present both the sports and the beyond-sports SO initiatives undertaken for people with ID.

## Methods

The material concerning SO’s new initiatives for this article was acquired from papers and research studies as well as from press, training, and conference materials. The official websites of the organization, both worldwide and national, were significant sources of information. *Special Olympics coaching development*

While between 2000 and 2010 the most important purpose was to increase the number of SO athletes around the world, at present, the main objective is professional training for coaches and sports instructors, aiming to provide people with ID with best conditions for development. Therefore, the model of a perfect coach was defined and it included the following qualities:

- A thorough knowledge of a given sports discipline and its rules;
- Professional communicative skills based on knowledge in the fields of special needs education, psychology, and the related sciences, all of which are indispensable while working with people whose abilities within the area are limited;
- Knowledge of medical problems that frequently accompany intellectual disabilities, such as obesity, cardiologic disorders, epilepsy, autism, orthopedic problems, visual impairment, hypacusia, etc.;
- Ability to identify optimal, individual goals for every competitor directed more often toward actual, short-term actions rather than toward long-term planning;
- Physical fittness to demonstrate the exercises and to frequently provide physical help to the least able participants;
- Organizational skills manifested in preparing and carrying out sporting competitions and participation in sports camps and sporting events;
- Ability to provide first aid (www.asep.com, 2014).

### Partnership with international (regional) sport federations

Creating a schedule of meetings and conferences with international sports federations is a very important element of the 2011–2015 Special Olympics Strategic Plan. The schedule makes possible the establishment of strategic partnerships in the areas of coaches’ development and expanding shared initiatives. This kind of calendar includes the participation of international sports federation leaders in the worldwide and continental Special Olympics Games as well as in international conferences.

The partnership with the Union of European Football Associations (UEFA), which was established in 1999 thanks to the initiative of SO Monaco, is a good example of such cooperation. The annual grants of around 250 000 euros provided by UEFA allow to organize a great number of European football initiatives such as tournaments, conferences, “SO European Football Week”, printing of training and promotional materials, as well as promotion during the Champions League in Lisbon in 2014. An excellent example of this cooperation was the SO football players’ exhibition match, which took place just before the quarter final of the EUFA European Championship in Warsaw in 2012.

The SO also cooperates with the International Basketball Federation Europe (FIBA Europe), what has led to significant progress in the development of basketball programs for disabled since 2004, when the Protocol of Collaboration was sealed in Rhodon (Greece). This cooperation resulted in the promotion of the SO during the European Basketball Championship in Serbia (2005), Poland (2009), Lithuania (2011), and Slovenia (2013).

In addition to this valuable cooperation with the FIBA, there is also a model cooperation with Euroleague Basketball, which was initiated in 2009. This cooperation enables a wide range of promotional campaigns during the Special Olympics Basketball Week, which takes place at the turn of November and December each year. The SO competitors have the opportunity to appear on the court beside the world’s best basketball players, what gives Euroleague the chance to promote the actions undertaken by the organization. Additionally, adhesive posters with the SO logo are on the boards surrounding the arena and on the court.

Another European federation that has cooperated with SO is the European Swimming League (LEN). They began their cooperation in 2005, and since 2006, the LEN has invited the SO competitors to take part in exhibition swimming races during the European SwimmingChampionship. The exhibition races have taken place twice on a long course pool in Budapest, in 2006 and in 2010, and on the short course pool in Helsinki, Rijeka, and Eindhoven. For the first time, the exhibition also took place in Poland during the European Swimming Championships in Szczecin in December 2011.

The SO bowling competitors have benefitted similarly from their partnership with the European Tenpin Bowling Federation, which started in 2009. The athletes were able to participate in the exhibition game during the World Championships in 2010 and then in the European Championships in 2011 in Munich, and in 2013 in Berlin.

The last Partnership Protocols were signed with the European Cycling Federation (UEC) in 2014 in Paris, the European Badminton Federation (BEC) in 2015 in Leuven and the European Athletics and Gymnastics Federation in Lausanne in 2011. The presidents of these federations will declare in the near future all sorts of promotional actions for the Special Olympics.

Signing of the collaboration proclamation with the International Federation of Adapted Physical Activity (IFAPA) by its president Yeshayahu Hutzler, and by the chairman of Special Olympics, Timothy Shriver, is also of great significance. This agreement opened the door for better communication and collaboration with a great number of organizations and universities on all continents (Coach Education Center, 2010).

### Sport Resources Teams (SRT)

Over 120 people in different countries help by offering their advice to the staff of the SO, what aims at developing better cooperation with SO competitors in different sport disciplines. They are prominent experts in 34 different disciplines, trained within the SO movement, and they frequently have strong links with national and international sports federations. They help voluntarily by examining the sports regulations and by participating in the process of producing coaching manuals. Additionally, they perform the role of fourth officials during important SO competitions. This group is called the Sport Resources Team for a particular discipline.

The Special Olympics movement attempts to gain an increasing number of such experts, aiming at developing more and more professional organizational skills (www.media.specialolympics.org).

### Extended quota for high level athletes

According to SO procedures, each national SO program receives a determined number of invitations to the world or continental games. Most often, the number of invitations is lower than the aspirations of such programs. However, a given country/SO program may receive an increased number of invitations for those competitors who significantly exceed the level of remaining SO athletes and who obtain their own individual achievements beyond the fixed limits.

For example, during the World Olympic Games in Athens in 2011, such extended quotas were offered to the best competitors in swimming who participated in the 400 m medley swimming competition and in the 1500 m open water swimming competition, in athletics for the 100, 800 and the 5000 m races, in the marathon and half marathon, in bowling, cycling, gymnastics, and golf. During the Winter Games in Korea in 2013, this type of “extended quota” was provided in cross-country and downhill skiing, speed and figure skating, and snowboarding. The SO extended quota program for outstanding athletes meets the coaches’ and parents’ expectations and, above all, gives the athletes a chance for real competition at the highest possible sporting level (Sport Rule Competitions, 2014).

### Athletes Leadership Program (ALPS)

The SO movement gives a lot of opportunities both to people with ID who have finished their careers as competitors and to those who have never been athletes. They empower these people, providing them with a chance to be close to their athlete friends and sometimes even to decide about the organization’s functioning. Therefore, they can be members of the national or regional SO management. In fact, for an organization to receive the official accreditation of the SO program, there is an obligation to include people with ID assisting with decisions. They can assist coaches, help in training units, look after the equipment, perform the warm-ups, etc. After proper training, people with ID may contribute as referees, assisting during competitions. A lot of them feel excellent in the role of a volunteer, carrying out different tasks during the competitions, such as serving meals and drinks, handing out medals, etc. Others, after adequate training, do well as speakers, representing their colleagues during official meetings and ceremonies. They are associated with a program called Global Messengers. Groups of reporters with disabilities have been also recently created in some countries with the purpose of commenting on their colleagues’ sporting competitions (Styczeń, 2009).

### Young Athletes

According to the regulations, the age of 8 years enables a person to take part in the SO competitions. Recently, however, a new program called Young Athletes has been established for participants aged 2.5 to 8 years old. At the same time, a “therapeutic”program has come about for parents, giving them the opportunity for close contact with other parents, exchanging experiences and becoming supporters and members of the SO Family Program in the future.

The Young Athletes Program is an extension of the early intervention program, which has been known for a long time in the rehabilitation of children with ID. Playing with the use of colorful, attractive equipment and with music significantly increases the children’s interest in sports. The purpose of this program is to arouse interest in sports through SO coaches conducting sports classes. The essence of the program is supporting motor and psychological development through stimulating physical activity. The program makes use of attractive and colorful, miniature but standard sports equipment. The training classes are based on developing various skills including marching, running, maintaining balance, jumping, clearing obstacles, throwing, catching, and kicking. The program is designed in such a way that the suggested sports activities engage youth participants in sports competitions (Styczeń, 2009; Special Olympics, 2014).

### Volunteering in schools

The SO organization is founded on the principle of volunteering. This large-scale development’s thriving around the world is possible thanks to volunteers. Undoubtedly, young people, including college and primary school students, are the core of the organization. Because of this, SO attempts to arouse these people’s interest and to educate them within this scope as early as possible. A special educational program called “Special Olympics Get into It” was created (Styczeń, 2009) with the basis of providing text books for teachers at ordinary schools to help them in conducting lessons connected with disabilities, acceptance, tolerance, and respect to every person, and, finally, with suggestions about helping the Special Olympics movement. The complexity of these text books is varied and adjusted to age at four educational levels, starting with early education and ending at the secondary school level. Research projects assessing the effects of conducting such actions showed considerable differences in these students’ everyday-life perceptions of people with ID. A great number of students have decided to become volunteers, and some of them have even taken part in full-scale educational activities and conferences for Special Olympics teenagers (Bogdan and Styczeń, 2004; Special Olympics, 2014).

### Unified Sports Program

The Unified Sports program intends to organize shared sports training sessions and competitions among teams consisting of fully-abled competitors and people with ID. This program contributes to integrating athletes with ID into society by providing them with the opportunity to interact with others and to make friends. It also contributes to the development of social skills and self-esteem.

#### Motor Activity Training Program

The Motor Activity Training Program (MATP), which is carried out by SO, is a unique sports program intended for people with profound ID, including people with infantile cerebral palsy. The program involves all people who, due to physical and intellectual limitations, do not have the opportunity to participate in other disciplines offered by SO. This program emphasizes the participants’ individual needs. It is focused on obtaining sports results based on the analysis of motor and intellectual abilities, indications of the special needs education, and physical movement needs that are underlain by physiotherapeutic rehabilitation. The MATP is executed most often at the level of special education where it correlates with physical education and a motor rehabilitation program as well as with subjects’ improving their self-care skills. Warm-ups, endurance development, both fine and gross motor skills and daily activities are part of the training program (Myśliwiec et al., 2005).

### Healthy Athletes

One of the greatest initiatives of the Special Olympics is the program called Healthy Athletes, which was introduced in 1996. Its mission is the improvement of physical fitness in people with ID and their better preparation to participate in practice and sports competitions. The Healthy Athletes program helps the SO competitors in maintaining health and the state of well-being at the highest level, and it facilitates achieving sporting success while improving the participants’ quality of life. The Healthy Athletes program consists of six subprograms (Dominik et al., 2007; Styczeń, 2009; Myśliwiec et al., 2009).

The longest existing program is the Special Smiles program. The examination of health and hygiene of the oral cavity was first carried out in 1993 during the sports competitions in Massachusetts, and in 1996 the program was incorporated into the Healthy Athletes program (Dominik et al., 2007; Turner et al., 2008; Styczeń, 2009).

Another program is the Healthy Hearing program. It aims at assessing the frequency of the occurrence of hearing impairments in athletes with different degrees of ID who take part in Special Olympics sports competitions (Starska and Łukomski, 2006; Dominik et al., 2007; Hild et al., 2009; Styczeń, 2009).

A program in which the SO competitors are subject to ophthalmological examinations is the Opening Eyes program, known in Poland by the name Healthy Eyes. Ophthalmologists, opticians, and optometrists are involved in the program and the following tests are conducted: visual acuity, oxyopia, the eyes’ alignment, color vision, and binocular vision. The program also includes an examination of auto refraction, a measurement of intraocular tension, an examination of the pupils, and ocular funds (Dominik et al., 2007; Styczeń, 2009).

The physiotherapists involved in the Healthy Athletes program participate in the FUN fitness program, which was introduced in 1998. It was established according to the suggestions of the American Physical Therapy Association, and the main focus is on assessing motor abilities, such as strength, balance, flexibility, and endurance (Dominik et al., 2007, Styczeń, 2009).

The Fit Feet program, in which the biomechanical efficiency and feet hygiene are evaluated, was incorporated into the Healthy Athletes project in 2003 by Patrick Nunan from the American Academy of Podiatric Sports Medicine. The first pilot study was carried out one year earlier in Ohio, USA, during the National Special Olympic Games. The aim of the Fit Feet program is the assessment of the SO participants’ lower extremities, paying special attention to feet and ankle articulation deformities, as well as to the analysis of the condition of the skin and nails (Dominik et al., 2007; Styczeń, 2009).

In Poland the shortest executed program is the Health Promotion program. This program is dedicated to educating the SO participants in respect to diet and negative effects of smoking, drinking alcohol, and using narcotic drugs (Dominik et al., 2007; Styczeń, 2009; Myśliwiec et al., 2009).

## Conclusion

All of the people connected with ID emphasize the necessity of carrying out the sports and beyond-sports programs of the Special Olympics. This group, including parents and care givers, has many times contended with the problem of exclusion, and this issue has been the driving force for social campaigns. Various forms of help have been organized as part of such campaigns. Sometimes the purpose is to provide immediate actions connected with activating people with disabilities in the community through organizing sports, cultural, and integrative events. Often this sort of help is of permanent nature, giving people with disabilities the opportunity to effectively function for a long time, even during their whole life (Marchewka, 2003).

A significant advantage and strength of SO programs consist in the fact that they have a country wide range, and finding them should not be hard for people with disabilities. The local, regional, and national levels enable continuity in the training process, providing, in the last stage, the opportunity to participate in large, frequently international sporting events organized abroad, often in very attractive locations.

It needs to be emphasized that the most important stage of work is performed at the local level, in local Sports Clubs, which are the basic functional units of the Olympics. The clubs implement basic principles of the organization’s mission. A research project carried out during the Special Olympics in 2005 indicated that first of all, participation in the Olympics programs gives people with disabilities the joy of winning medals, it creates the opportunity to demonstrate courage, and it allows them to take part in sporting competitions. People permanently connected with the SO emphasize the sense of satisfaction experienced while conducting these sports activities. However, they also bring forward the competitors’ behavior, their difficulties in communication, and multifaceted health problems (Conatser et al., 2009). Participation in the SO competitions has also been proven to increase self-esteem and improve the physical fitness of the people with disabilities (Marchewka, 1998). Special Olympics supports the quality of life of people with ID. Parents of athletes with disabilities have indicated that the organization makes it possible for them and their children to overcome the feeling of isolation and pessimism. It improves relations between the parents and their children with disabilities and improves the parents’ preparation to look after their children (Weiss, 2008).

Even though the strong position and positive aspects of SO actions are commonly known, a frequent opinion arises that there are no substantial research projects that emphasize their social function. The exploration of progress, referring to the sporting aspect as well as to the functioning not connected with sports, would provide the opportunity to observe changes taking place within this population. Therefore, taking up scientific projects connected with the broadly defined activities of SO should contribute to improving the participants’ functioning and quality of life.
